# Sequential Organ Failure Assessment predicts outcomes of pulse indicator contour continuous cardiac output-directed goal therapy: A prospective study

**DOI:** 10.1097/MD.0000000000008111

**Published:** 2017-09-29

**Authors:** Wei Zhang, Quzhen Danzeng, Xiaoting Feng, Xing Cao, Weiwei Chen, Yan Kang

**Affiliations:** a Department of Critical Care Medicine, Sichuan University West China Hospital, Chengdu, Sichuan; b Department of Critical Care Medicine, Affiliated Hospital of Zunyi Medical College; c Department of Critical Care Medicine, First People's Hospital of Zunyi, Zunyi, Guizhou, China.

**Keywords:** prognosis, sepsis, septic shock, SOFA

## Abstract

According to the new sepsis definitions, septic shock is defined as a subset of sepsis in which the underlying circulatory and cellular/metabolic abnormalities are profound enough to substantially increase mortality. We evaluated the predictive efficacy of the Sequential Organ Failure Assessment (SOFA) score in critically ill patients with septic shock undergoing pulse indicator contour continuous cardiac output (PiCCO)-directed goal therapy (PDGT).

We conducted a single-center, prospective, observational study of 52 patients with septic shock undergoing PDGT. The putative prognostic factors, including the severity scores (SOFA and Acute Physiology and Chronic Health Evaluation II [APACHE II] scores), were analyzed within 24 hours after diagnosis of septic shock. We assessed and compared the predictive efficacy of risk factors for 28-day mortality of patients with septic shock undergoing PDGT.

Among the patients with septic shock undergoing PDGT, the SOFA scores of nonsurvivors were significantly higher than those of survivors (*P* < .001); the area under the receiver operating characteristics curve was higher for SOFA than for APACHE II (*P* = .005). The outcomes of the logistic regression analysis for 28-day mortality showed that the odds ratio, 95% confidence interval, and *P*-value of SOFA were 1.6, 1.2 to 2.1, and <.001, respectively.

The predictive model of the SOFA score is able to accurately predict the outcomes of critically ill patients with septic shock undergoing PDGT.

## 1 Introduction

In 2015, the Sequential Organ Failure Assessment (SOFA) criteria became the cornerstone of the new sepsis definition (Third International Consensus Definitions for Sepsis and Septic Shock [Sepsis-3]), replacing the systemic inflammatory response syndrome criteria (Sepsis-2).^[1]^ In the new definition, sepsis is divided into only 2 subgroups: sepsis and septic shock, leaving 2 subgroups of severe sepsis and septic shock in the old definition and excluding the subgroup of sepsis. Septic shock is still a major cause of intensive care unit (ICU) admission and death.^[2]^ Because the results of early goal-directed therapy (EGDT) in critically ill patients with septic shock seemed to be unfavorable in several recent clinical trials and meta-analyses,^[3–7]^ few clinical trials of EGDT combined with pulse indicator contour continuous cardiac output (PiCCO) monitoring^[8,9]^ have been carried out. Compared with Swan–Ganz catheterization monitoring, PiCCO monitoring, as one of the selected monitoring instruments for critically ill patients with septic shock, is associated with a lower risk of complications and death.^[10]^ Especially in recent years, PiCCO monitoring has been more widely used in critical care medicine than Swan–Ganz catheterization monitoring. By combining PiCCO with other monitoring instruments, including arterial blood gas analysis and bedside ultrasonography, precise medical treatment for hemodynamic support in critically ill patients with septic shock can gradually become a reality.

SOFA was established by the European Society of Intensive Care Medicine (ESICM) in October 1994 as a severity metric for organ dysfunction in critically ill patients.^[11]^ Since then, it has been widely used in critical care medicine in combination with the Acute Physiology and Chronic Health Evaluation (APACHE) system^[12]^; the 2 scores complement each other, thus greatly contributing to studies on critical illness.^[13–19]^ Although the intention of the original design was not to evaluate the prognosis of critically ill patients, the SOFA score has in fact shown a promising relationship with the prognosis of critically ill patients. In particular, several studies have focused on the prognosis of critically ill patients with septic shock.^[20,21]^ However, whether SOFA can predict the prognosis of critically ill patients with septic shock using PiCCO-directed goal therapy (PDGT) remains unclear. The aim of the present study was to determine the prognostic value of SOFA in critically ill patients with septic shock undergoing PDGT.

## 2 Methods

### 2.1 Study design

The present study was a single-center, prospective, observational study.

### 2.2 Setting

The study was conducted in an adult ICU of an 1800-bed tertiary care hospital (Third Affiliated Hospital of Zunyi Medical College or First People's Hospital of Zunyi, Guizhou, China). The study was approved by the Ethics Committee of First People's Hospital of Zunyi, and written informed consent was obtained from each patient's next of kin or a surrogate decision-maker from June 1, 2015 to December 31, 2015. The trial was registered at clinical trial.gov ChiCTR-OOC-15006338.

### 2.3 Patients

Patients with septic shock were selected from all patients consecutively admitted to the ICU of First People's Hospital of Zunyi during the clinical trial.

### 2.4 Definition

Sepsis is defined as life-threatening organ dysfunction due to a dysregulated host response to infection. The clinical criteria for sepsis include a suspected or documented infection and a proxy for organ dysfunction (i.e., SOFA scores acute increase of 2 or more in SOFA score system).^[10]^ Septic shock is defined as a subset of sepsis in which the underlying circulatory and cellular/metabolic abnormalities are profound enough to substantially increase mortality. The clinical criteria for septic shock include sepsis and the need for vasopressor therapy to increase the mean arterial pressure to >65 mm Hg and the lactate concentration to >2 mmol/L after adequate fluid resuscitation.

### 2.5 Inclusion criteria

Patients who met the following criteria were included in the study:

1) Age of ≥18 years
2) ICU stay of ≥24 hours
3) No missing data
4) Informed consent to participate in the study.

### 2.6 Screening flowchart

**Figure 1:**
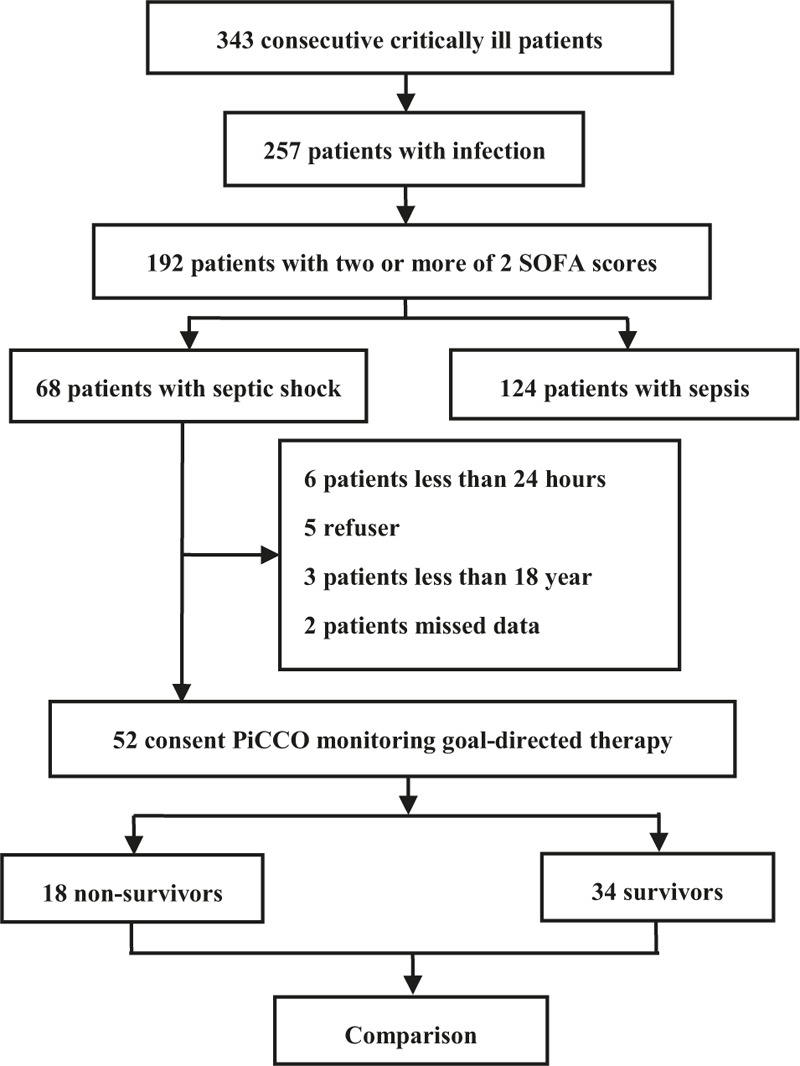
Screening flowchart.

The screening flowchart appears in Fig. 1. We selected and enrolled critically ill patients with septic shock undergoing PDGT.

### 2.7 Flowchart of PDGT

**Figure 2:**
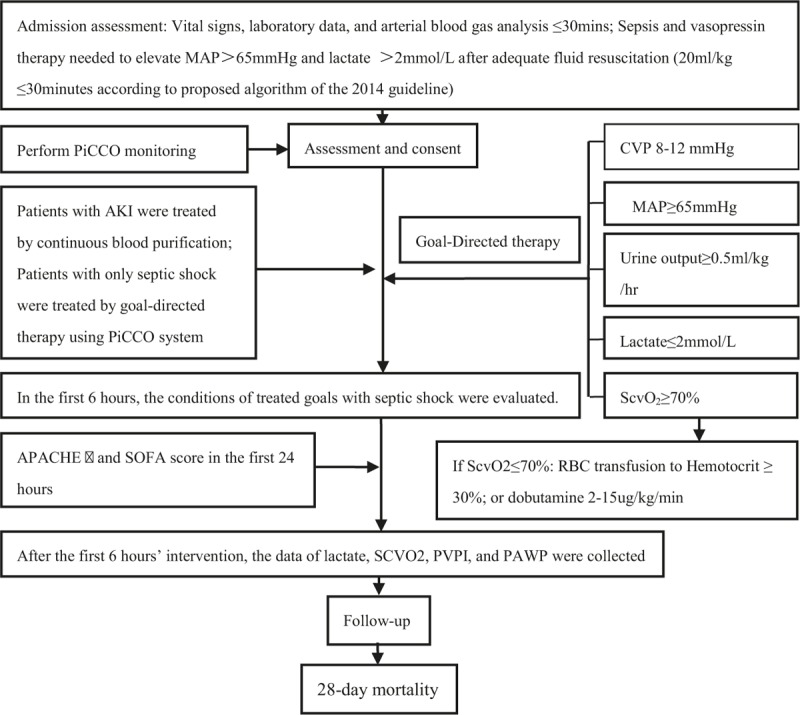
Experimental operation flowchart.

The flowchart of PDGT appears in Fig. 2. In this study, PDGT in critically ill patients with septic shock was dynamically assessed in real time to obtain the quantitative cardiac preload and postload, myocardial contractility, extravascular lung water index (EVLWI), pulmonary vascular permeability index (PVPI), and central venous oxygen saturation (ScvO_2_) according to the intervention requirements. This was combined with measurement of the serum lactate concentration, which can prevent and minimize occurrence of fluid overload or insufficient capacity and help to select appropriate vasopressors in the process of resuscitation.

Goal-directed therapy in the first 6 hours is essential for critically ill patients with septic shock. We assumed that the response of patients who underwent resuscitation in the first 6 hours was likely to affect the outcomes of septic shock; thus, we used the parameters at the 6th hour after resuscitation in the analysis. All therapeutic interventions were based on the proposed algorithm for the treatment of septic shock according to the established guidelines on septic shock.^[22]^

The SOFA and APACHE II scores on admission were evaluated within 24 hours of admission; for those patients staying in the ICU in whom septic shock occurred, the evaluation was performed within 24 hours after the diagnosis of septic shock. The 28-day mortality after enrollment was regarded as the outcome variable. The outcomes were divided into 2 cohorts according to the type of outcomes: the survivor and nonsurvivor cohort.

### 2.8 Data collection

The following clinical data on the enrolled patients were collected and analyzed: demographics, site of infection, length of ICU stay, duration of mechanical ventilation, percentage of mechanical ventilation and continuous renal replacement therapy, and parameters at the 6th hour after resuscitation (lactate concentration, PVPI, ScvO_2_, urinary output [UO], and clinical outcomes [28-day mortality]). The APACHE II and SOFA scores were calculated based on the worst physiologic parameters^[12,23]^ prompting either admission to a stay in the ICU within 24 hours after the diagnosis of septic shock.

### 2.9 Bias

Those who participated in the data collection for the study were blind to the study design, and the study designers did not participate in the data collection.

### 2.10 Statistical analysis

Categorical variables, expressed as rates (%), were compared using the *χ*^2^ test. Data were checked for a normal distribution using the Kolmogorov–Smirnov test. Quantitative data with a skewed distribution are summarized as median and interquartile range and were assessed using the nonparametric Mann–Whitney test. Quantitative data with a normal distribution are expressed as mean ± standard deviation, and comparisons between groups were carried out with Student *t* test for continuous variables. Receiver operating characteristic (ROC) curves were plotted to assess the SOFA score and other variables in terms of their diagnostic and prognostic capabilities in patients with septic shock undergoing PDGT. Multivariate analysis of the clinical variables was carried out by logistic regression. Pairwise comparison of the ROC curves between the SOFA score and other variables was performed using MedCalc Empower Stats statistical software (version 15.8). All statistical tests were 2-tailed, and a *P*-value of <.05 was considered significant.

## 3 Results

All patients who were consecutively admitted to the adult ICU were screened (n = 343); 257 (74.9%) of them had proven or documented infection. Among these 257 patients with infection, 192 (56.0%) were diagnosed with sepsis. According to the clinical criteria of Sepsis-3, 124 (36.2%) patients were diagnosed with sepsis and 68 (19.8%) with septic shock. Among the 68 patients with septic shock, 16 were excluded from the study for various reasons: a <24-hour ICU stay (n = 6), refusal to join the study (n = 5), age of <18 years (n = 3), and missing data (n = 2). In total, 52 patients with septic shock undergoing PDGT were finally included in the analysis (Fig. 1).

### 3.1 Baseline characteristics

The patients were divided into 2 cohorts based on the 28-day mortality: the survivor and nonsurvivor cohort (Table 1). The nonsurvivor cohort comprised 18 (34.6%) patients. The mean APACHE II score, SOFA score, and 6th-hour parameters such as the lactate concentration, PVPI, EVLWI, and ScvO_2_ were lower in the survivor than nonsurvivor cohort (*P* < .05). However, the mean UO and length of stay in the ICU were higher in the survivor than nonsurvivor cohort (*P* < .05). The mean mechanical ventilation duration and other variables were not significantly different between the 2 cohorts.

**Table 1:**
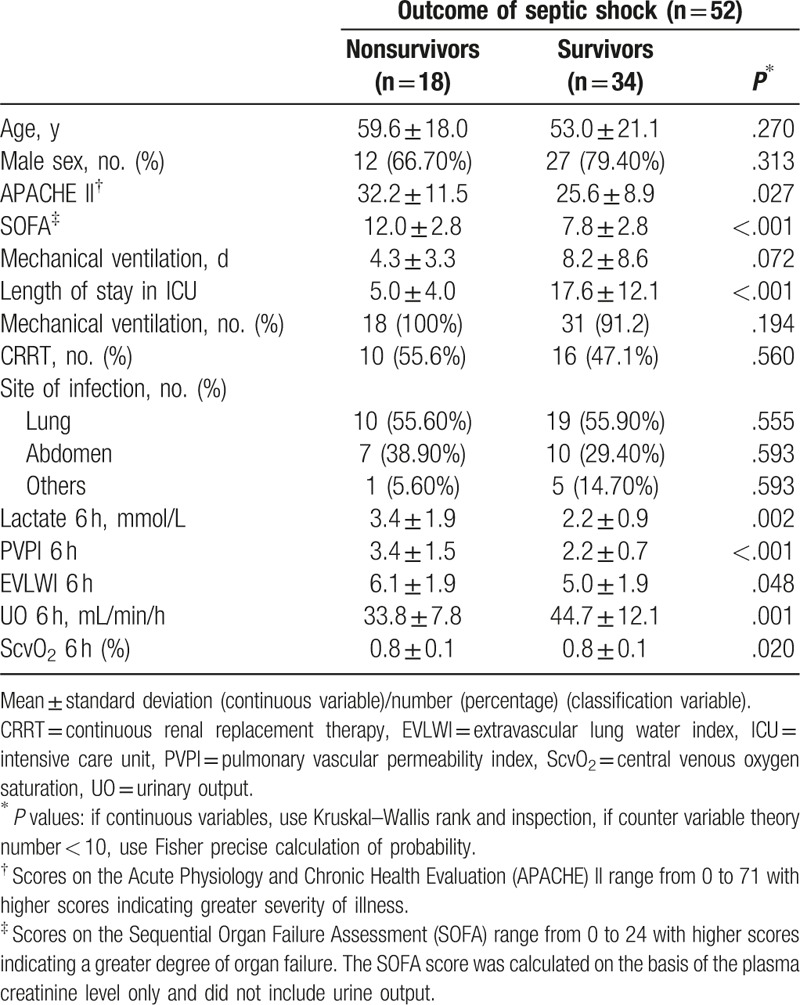
Baseline characteristics of the survivors and nonsurvivors based on 28-day mortality.

### 3.2 Logistic regression analysis

Predictors of 28-day mortality (n = 18) according to the logistic regression analysis of patients with septic shock undergoing PDGT are shown in Table 2. The following variables were included in the multivariate logistic regression: age, sex, length of ICU stay, duration of mechanical ventilation, lactate concentration, PVPI, EVLWI, ScvO_2_, UO, and SOFA and APACHE II scores within 24 hours after the diagnosis of septic shock. The odds ratios of the SOFA and APACHE II scores, lactate concentration, PVPI, ScvO_2_, and UO were 1.6, 1.1, 1.4, 1.4, 1.1 × 10^–5^, and 0.82, respectively, indicating that these parameters were prognostic risk factors in patients with septic shock undergoing PDGT.

**Table 2:**
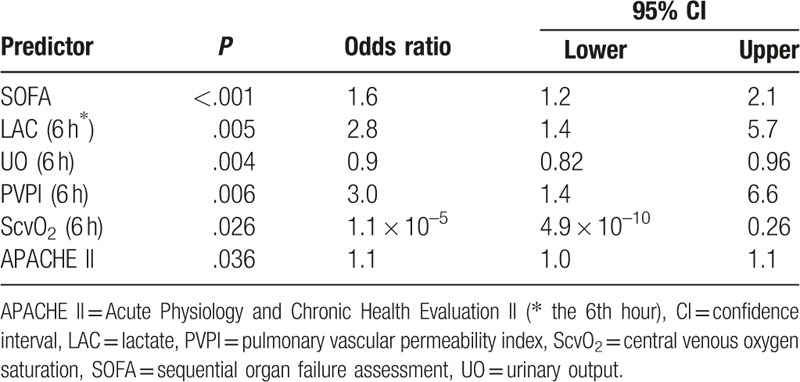
Logistic regression analysis of possible risk factors for septic shock prognosis.

### 3.3 Prognostic value of SOFA and other variables

**Figure 3:**
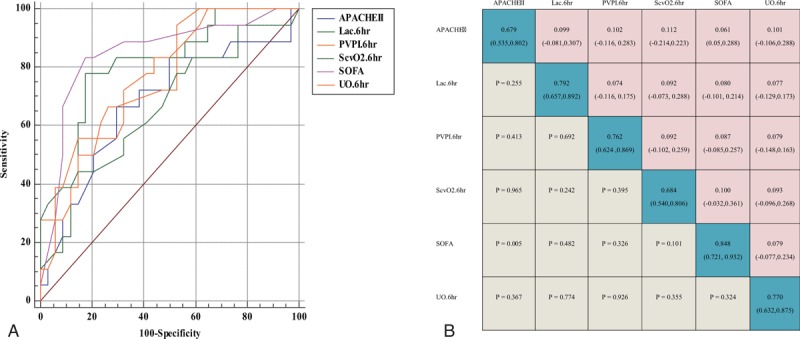
Receiver operating characteristic curves and area under the receiver operating characteristic curves.

ROC analysis was used to evaluate the prognostic value of the SOFA score and other variables in patients with septic shock undergoing PDGT. For the septic shock prognosis, the areas under the ROC curve for the SOFA score, APACHE II score, lactate concentration, PVPI, ScvO_2_, and UO were 0.848, 0.679, 0.792, 0.762, 0.684, and 0.770, respectively, indicating that SOFA had high predictive value in the prognosis of septic shock in patients undergoing PDGT (Fig. 3).

### 3.4 Pairwise comparison of area under the ROC curves

Compared with the APACHE II system, the results of the data analysis suggested that SOFA was a favorable predictor of evaluating the prognosis in critically ill patients with septic shock undergoing PDGT (*P* < .05). However, compared with other variables such as the lactate concentration, SOFA score, UO, PVPI, and ScvO_2_, the differences were not statistically significant (Fig. 3).

## 4 Discussion

The clinical criteria of the new sepsis definition based on the SOFA criteria not only provided an updated definition and diagnostic criteria for septic shock but also made clinical studies more convenient.^[1]^ Although several very well-designed and high-quality studies have shown negative outcomes of EGDT in patients with septic shock,^[4–6]^ the concept of EGDT still more or less dominated the selection of therapeutic strategies among clinicians. The emergence of PiCCO technology allowed for a greater understanding of the hemodynamic laws of septic shock. PiCCO technology can make precision medical treatment a reality when providing hemodynamic therapy to critically ill patients with septic shock, especially in the evaluation of cardiac preload and postload, myocardial contractility, EVLWI, PVPI, and oxygen delivery and consumption. The application of PiCCO can minimize the occurrence of fluid overload and insufficient capacity as well as reduce unnecessary intervention with vasopressors and therefore decrease the risks of their side effects. PDGT may provide a new direction in the treatment of septic shock. The objective of this study was to evaluate the prognostic value of the SOFA score in critically ill patients with septic shock undergoing PDGT. We found that the predictive model of the SOFA score can accurately predict the outcomes of such patients.

PiCCO did not become a routinely used clinical monitoring tool in critically ill patients with septic shock. Some studies have shown poor outcomes of septic shock under PiCCO monitoring compared with central venous pressure-based fluid management.^[24]^ We speculated that those results were related to both insufficient training in PiCCO technology and incomprehensive hemodynamic knowledge among the clinicians who administrated the technology; as a result, they acted with insufficient confidence when making selections according to the PiCCO monitoring parameters. In our ICU, PiCCO technology had been used in critically ill patients with septic shock for >4 years before the present study was begun. More importantly, all clinicians in our department were required to undergo rigorous and standardized training on hemodynamic and PiCCO monitoring technology. The SOFA score, as a severity metric for critical illness, is theoretically related to the prognosis of critically ill patients with septic shock^[25,26]^; our study proved this point. In particular, the SOFA score showed good prognostic value in patients with septic shock undergoing PDGT, and this can help to achieve accurate prognostic evaluation after establishing the diagnosis of septic shock in the first 24 hours of ICU admission.

### 4.1 Strengths

The study was innovative in that it was performed in the context of establishing a new definition of sepsis based on the SOFA criteria and the failure of EGDT in clinical trials. PiCCO technology has not only increased our understanding of the hemodynamic laws of septic shock, but it has also increased the awareness and use of EGDT (termed PDGT in the present study). Moreover, because the SOFA criteria have become the cornerstone of the new definition of sepsis, the prognostic value of the SOFA score based on the updated definition of septic shock requires reevaluation through clinical trials. Although the critically ill patients with septic shock undergoing PDGT in our study only accounted for 15.2% (52/343) of all patients admitted to the ICU, the results of this study are still significant with respect to the new sepsis definition.

### 4.2 Limitations

This study also has several limitations. First, it was a single-center clinical trial of adult patients; thus, the study population was not representative. Second, the study duration was only 6 months (July–December 2015), which may have produced selection bias due to seasonal effects because the year-round population was not covered. Third, the sample size was small due to the limited time and site of the trial. Finally, few clinicians in medicine are practicing PDGT.

### 4.3 Generalization

The advantages of PDGT over EGDT for patients with septic shock remain unclear. However, we identified the advantages of the SOFA criteria in a predictive model of 28-day mortality for critically ill patients with septic shock undergoing PDGT and progressively proved the availability of clinical criteria for the new definition of septic shock. Our study also revealed an interesting phenomenon: The indicators at the 6th hour of resuscitation, such as the lactate concentration, PVPI, ScvO_2_, and UO, were equivalent to the SOFA scores in terms of reflecting the prognosis of patients with septic shock. In future studies, we will attempt to integrate these indicators to create a new evaluation system through application of various statistical methods such as a monogram to more fully assess the prognosis of critically ill patients with septic shock.

## 5 Conclusions

The herein-described predictive model of the SOFA criteria is able to accurately predict the outcomes of critically ill patients with septic shock undergoing PDGT.

## Acknowledgments

This prospective observational study was approved by the ethics committee of First People's Hospital of Zunyi City in Guizhou, China. The authors thank Professor Craig. M. Coopersmith, Chairman of the Society of Critical Care Medicine in the United States, for providing us the new definition of sepsis (Sepsis-3) in the 9th Congress of the Chinese Society of Critical Care Medicine on May 21, 2015 in Shanghai, China. We also thank Changzhong Chen from the Dana-Farber Cancer Institute of Harvard Medical School for assisting with the statistical analysis and data management. Finally, we thank all doctors, including Zhao Wang, Chuanjie Xu, Yang Yang, and others, for assisting with the data collection in our study.
